# Effects of Stochasticity in Early Life History on Steepness and Population Growth Rate Estimates: An Illustration on Atlantic Bluefin Tuna

**DOI:** 10.1371/journal.pone.0048583

**Published:** 2012-10-31

**Authors:** Maximilien Simon, Jean-Marc Fromentin, Sylvain Bonhommeau, Daniel Gaertner, Jon Brodziak, Marie-Pierre Etienne

**Affiliations:** 1 AgroParistech-ENGREF (École Nationale du Génie Rural des Eaux et des Forêts), Paris, France; 2 UMR 212 EME (Exploited Marine Ecosystems), IFREMER (Institut Français de Recherche pour l’Exploitation de la mer ), Sète, France; 3 UMR 212 EME (Exploited Marine Ecosystems), IRD (Institut de Recherche pour le Développement), Sète, France; 4 Pacific Islands Fisheries Science Center, Honolulu, Hawaii, United States of America; 5 UMR 518 AgroParistech-INRA (Institut National de Recherche Agronomique), Paris, France; Aristotle University of Thessaloniki, Greece

## Abstract

The intrinsic population growth rate (*r*) of the surplus production function used in the biomass dynamic model and the steepness (*h*) of the stock-recruitment relationship used in age-structured population dynamics models are two key parameters in fish stock assessment. There is generally insufficient information in the data to estimate these parameters that thus have to be constrained. We developed methods to directly estimate the probability distributions of *r* and *h* for the Atlantic bluefin tuna (*Thunnus thynnus*, Scombridae), using all available biological and ecological information. We examined the existing literature to define appropriate probability distributions of key life history parameters associated with intrinsic growth rate and steepness, paying particular attention to the natural mortality for early life history stages. The estimated probability distribution of the population intrinsic growth rate was weakly informative, with an estimated mean r = 0.77 (±0.53) and an interquartile range of (0.34, 1.12). The estimated distribution of *h* was more informative, but also strongly asymmetric with an estimated mean h = 0.89 (±0.20) and a median of 0.99. We note that these two key demographic parameters strongly depend on the distribution of early life history mortality rate (*M_0_*), which is known to exhibit high year-to-year variations. This variability results in a widely spread distribution of *M_0_* that affects the distribution of the intrinsic population growth rate and further makes the spawning stock biomass an inadequate proxy to predict recruitment levels.

## Introduction

Bayesian state-space modeling is now developing into a practical approach for stock assessment studies and appears particularly adapted for fisheries management because it provides a statistically rigorous framework for deriving quantitative estimates for decision analyses [1], [2]. Bayesian models require specification of prior probability distribution functions (pdf) for model parameters; posterior probability distributions are derived from the combination of prior information and the sample likelihood information contained in the data. This sequential learning process allows for the incorporation of expert and biological knowledge into the prior pdf and the use of informative priors can improve inference by multiplying the available information sources [3]. The approach also allows the use of additional and independent information from different sources that is usually ignored within traditional stock assessment models. The sequential learning principle also makes possible to connect the stock assessment model to sub-models in a coherent statistical framework (e.g [4], [5]). Overall, the Bayesian framework has been applied to many different exploited fish stocks, such as salmon [2], tuna [6], rockfish [7], small pelagics [8] and sharks [9].

The aim of the present study was the elicitation of prior distributions for the steepness parameter of the stock-recruitment relationship (SR) and for the intrinsic population growth rate of the biomass dynamic model for the Atlantic bluefin tuna (ABFT, *Thunnus thynnus thynnus)*. Although the stock assessment for this species is traditionally conducted using VPA-ADAPT method [10], [11], biomass dynamics models are currently used for several tuna stocks and may be seen as an interesting alternative. Estimates of steepness are needed when using VPA, in order to estimate MSY base reference points and to conduct stock projections; also the use of integrated assessment models which can require the estimation of thousands of parameters need priors for key parameters such as steepness.

The steepness parameter that measures the decrease in recruitment which would occur if spawning potential is 20% of its unfished level [12]–[14] is now widely used to fix the SR relationship (several scenarios are commonly run with different values of steepness). The population growth rate of the logistic equation [15] is one of the parameter for which an informative prior is usually considered for the biomass dynamic model [16] formulated as a state-space model [6]. These two demographic parameters are strongly constrained in most of fisheries stock assessment models. They further have a strong impact on the estimation of the Maximum Sustainable Yield (MSY) and on the outcomes of the projections used to determine future Total allowable Catch (TAC). Consequently, the choice of constraints or prior distribution strongly influence the outputs of models, see for instance [17]–[19].

Informative priors for demographic parameters of marine population were first deduced from meta-analysis, see e.g. [1], [20] for the steepness of demersal species and [6], [21] for the population growth rate of various groups of bony fish. Informative priors have also be elicited using available knowledge on biology and reproductive ecology. In age-structured population models, functional relationships allow the calculation of the population growth rate from life history parameters [9], [22], [23]. More recently, the estimation of the probability distribution function of the steepness has been proposed by [24] using life history information. More specifically, the steepness is expressed as a function of the recruitment per unit of parental biomass and the biomass per recruit, which are themselves expressed as functions of vital rates.

Among vital rates, Mangel et al approach requires the specification of the fecundity at age in absolute number and natural mortality rate at age. The latter is often fixed based on a variety of assumptions (e.g. natural mortality is derived from this of the southern bluefin and assumed to be age-dependent for the eastern stock of Atlantic bluefin, while it is fixed at 0.14 for the Western stock, [25]). However, there are serious issues concerning the estimation of these quantities for bony fish, specifically during early life period [26], [27]. Therefore, most studies proposing informative prior distribution for population growth rate of bony fish circumvent the problem of young-of-the-year mortality rate (YoY) by using a SR relationship, (e.g. [6], [28]). Doing so, the fecundity of age-group is expressed in number of recruits and the knowledge of mortality rate of the stages before recruitment is no longer necessary. Other studies focused on marine species for which we have a relatively better knowledge of fecundity and YoY survival: e.g. sharks [9], [29], [30], sea turtles [31], marine mammals [32]. While [32] raised the problem of the juvenile survival in prior elicitation for the population growth rate of marine mammals; most studies on fish population using priors for demographic parameters did not take into account uncertainties around the SR relationship or around YoY survival. Recruitment process is known, for a long time, to be complex and stochastic [33] and as a consequence the survival rates of early life stages remain difficult to quantify [34]. This issue has been intensively debated and studied since Hjort's pioneering work [35], [36] and has to be examined when using biological assumptions constraining key demographic parameters of exploited fish populations.

The present study aims at assessing how uncertainty about vital rates during earliest life stage affects the probability distribution steepness and population growth rate. To do so, we: (*i*) estimate uncertainties on vital rates of ABFT from hatching to maximum age with an emphasis on the pre-recruit mortality, (*ii*) include these uncertainties in the estimation of the probability distributions of the steepness and population growth rate and (*iii*) discuss the consequences of a high variability in the early life stages survival on those two key parameters.

## Materials and Methods

### Outline of the General Methodology

We focus on the determination of the probability distribution functions for some vital rates required to estimate the steepness and the growth rate of a fish population. These vital rates encompass mortality of pre-recruit stages, natural mortality at each age after recruitment and absolute annual fecundities at age. Working in a Bayesian framework, these vital rates are modeled using random variables. An extensive examination of the existing literature on the biology and ecology of bluefin tuna and tuna species in general has been carried out in order to define appropriate distributions for those random variables and is described in the following.

In the framework of age-structured population models, steepness and population growth rate have been expressed as functions of vital rates (fecundities and mortality rate at ages), using the demographic methods proposed by [9] for the population growth (r) and [24] for the steepness (h). Even if they are described as a function of vital rates, their exact distributions can't be obtained analytically and we use a Monte-Carlo simulation method to construct empirical probability distribution for *r* and *h* [37], [38], i.e. random samples were drawn in the vital rate pdf's, steepness and population growth rate are then calculated for each drawing and hence their empirical probability distributions were derived.

### Population Growth Rate

We used Leslie population model [23] to compute the population growth rate. This classical approach has been widely used in ecology [39] and is one of the demographic methods reviewed by [9] to elicitate the prior of the population growth rate. The population is described by *N(t)* vector of length *A* describing the number of individuals in each age-class at time t, with terminal age *A* (number of age groups). A transition matrix T determines the contribution of each individual to the next age-group and to the new generation. The entries of the Leslie matrix are *S(i)* and *F(i)*: *i.e*., the survival rate from age i to age i+1 and the fecundity at age i (*i.e.* the average number of age zero female individuals produced by an individual), respectively. In the matrix form, the model is written in the recurrence relationship (1):
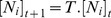
(1)


As *T* coefficients are all positive and constant over time, the composition of the population at *t*+*n* can be predicted by (2):
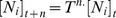
(2)


As t tends to infinity, the system reaches equilibrium and the contribution of each age group in total population becomes stable. The population growth rate, *r*, is *r = ln(λ)* with *λ* is the dominant eigenvalue of matrix *T* [39]. In our Monte Carlo approach, *r* is computed for 10000 Leslie population matrices resulting from 10000 random realizations in the pdf parameters.

### Steepness Parameter for the Beverton-Holt Stock-Recruitment Relationship

[24] and [21] expressed the steepness in a Beverton & Holt stock-recruitment model, such as :
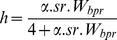
(3)where α is the maximum number of recruits per spawning biomass unit (slope of SR curve when spawning biomass approaches 0), *sr* the sex-ratio and the expected surviving biomass per recruit. *W_bpr_* is the expected surviving biomass per recruit (SPR*_F = 0_* in [21]) given in Eq. 4 where *l*(*a*) is the fraction of individuals surviving from recruitment (age 1) to age a, the weight of a female at age a, and *g*(*a*) the probability that a female is mature at age a



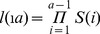
(4)

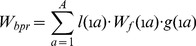
(5)


The simulation of an age-structured population is a necessary step for the calculation of α to avoid the use of a stock-recruitment relationship (as in [21]) because such as relationship remains, for ABFT, unknown and very poorly fitted from stock assessment data (see [25], [40]). We used the approach by [24] to generate a population of N individuals with an age-structured model defined by the vector of mortality rates. Once the population is simulated each individual’s age is known and its corresponding life history traits (length, weight, fecundity, probability of being mature) can be estimated. Then, α is given by the ratio of the number of surviving recruits (e^−M*0*^.F*_sim_*), where M_0_ is the cumulative mortality from fertilization to age 1 (Eq. 1) over the female spawning biomass simulated (B*_sim_*). The number of surviving recruit is obtained using Eq. 6, with F*_sim_* being the total number of oocytes produced by the simulated population.
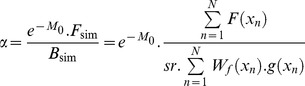
(6)where *x_n_* is the age of the n^th^ fish, *F(x_n_)* the number of age zero female individuals produced by this age-N individual, *g(x_n_)*, its probability of being mature, *W_f_(x_n_)*, its weight. It is possible to write explicitly *h* as a function of *M_0_*,



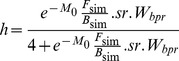
(7)Steepness sample is computed from random realizations in vital rate pdf. To generate a sample for α we repeated the simulation for K = 1000 population of N individuals. 1000 values of *W_bpr_* are computed from Eq. 5.

In this approach, the estimations of *r* and *h* assumed independence between the random variables that are involved in their calculations, which may result in overestimating their variability. As the potential correlation between *M_0_* and fecundity is unknown, we did a sensitivity analysis to test the influence of potential correlations among these variables, using a conservative approach. To do so, we re-estimated *r* and *h* distributions by implementing a bivariate random sampling procedure based on the empirical distributions of *M_0_* and fecundity, using a correlation coefficient of 0.7 and 0.9 between both variables. This procedure allowed us to re-estimate the distributions of *r* and *h* that were expected to be less variable than distributions obtained under the assumption of independence.

### Estimation of the Young of the Year Mortality Rate

#### Modeling approach

The mortality rate of the Young of the Year, *M_0_*, is assumed to be the sum of the mortalities resulting from non-fertilization of the eggs *M_f_*, hatching *M_h_*, and cumulative mortalities in the early stages (larvae and juveniles) *M_y_* (Eq. 8). For Atlantic bluefin tuna, this cumulative mortality is assumed to occur over the 180 days following the peak of the spawning season that takes place in June [41], [42]. As a consequence, *M_y_* is the sum of daily mortality rates *M_d_(x)*, where *x* is the number of days after fertilization, from the 1^st^ of July to the end of the calendar year (Eq. 9). *M_y_* takes into account both density-dependent and density-independent mortality rates, such as death by predation (including cannibalism), competition, starvation, disease, or hostile/favorable environmental conditions.
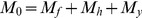
(8)

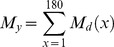
(9)


Quantifying the mortality of tuna early life stages from field’s experiments is a challenging and difficult task. Nonetheless, there were a few attempts over the last two decades (Table 1), through larval surveys [43]–[46]. However, these observations are not consistent among the different surveys, possibly because they display very high day-to-day variations : from 0.06 to 2.75 days^−1^ according to [46]. Therefore, they can hardly be used to quantify the overall mortality of young of the year. To overcome this issue, we used the same approach as [24] and estimated the daily mortality of the larvae and young juveniles through the equations established by [47]:
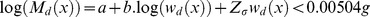
(10)

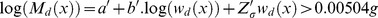
(11)where a, a', b and b' are parameters derived from McGurk’s log-regression [47] and the individual dry weight x days after fertilization. *Z_σ_* and *Z_σ'_* are the estimated variances around log-regressions which are assumed to be random variables normally distributed with mean zero and standard deviation σ and σ'.

**Table 1:** Instantaneous and cumulative mortality rates of YOY tunas and small pelagic species. Age is indicated in days post-hatching or days post exogenous feeding (dpef).

Species	Age	Mortality	Reference
		*Inst. mortality rate (day^−1^)*	
*Thunnus thynnus*	3 to 10	0.2	Scott et al. (1993)
*Thunnus albacares*	3 to 14 dpef	0.16	Lang et al. (1994)
		0.41	
*Thunnus maccoyii*	11	0.68	Davis et al. (1991)
	12	0.97	
*Thunnus orientalis*	5	1.66	Satoh et al. (2008)
	6	2.41	
	7	2.75	
	8	0.06	
	9	1.74	
	10	NA	
	11	1.52	
	12	1.52	
		*Cumul. mortality rate*	
*Scomber scombrus*	11.42	6.02	Ware and Lambert (1985)
	17.3	8.14	
*Engraulis encrasicolus*	100	5.99	Allain et al. (2007)
	100	6.5	
*Engraulis encrasicolus*	180	9.94	Pertierra et al. (1997)
*Engraulis mordax*	180	9.56	Lo et al. (1995)
*Sardinops sagax*	180	12.25	
*Sardinops caeruleus*	180	7.88	
	180	8.465	

#### Data used for parameterization

As shown in Eq. 1, the YoY mortality is split into fertilization rate, hatching rate and daily mortality up to 180 days after hatching. Fertilization rate of teleost species notably depends on sperm quality and mating behavior [48]–[50]. Although most tuna species exhibit particular mating behavior with male chasing the females [51], external fecundation and the very large number of expelled gametes makes fertilization rate of large pelagic fishes difficult to measure. Hatching rate for tuna is reported at about 80% [52], [53] and "normal" hatching, *i.e.* without lethal malformations between 40 and 60% [53]. Following these studies, we assumed that fertilization and hatching are responsible for a 50% loss of expelled oocytes and more precisely to be a random variable normally distributed with mean of -log(0.5). Considering the lack of precision and unknown variability of these biological processes a CV of 10% was used for this parameter. Note that a CV of 25% has been also tested for a few life history parameters (i.e. for non-fertilization, and hatching rate and post-recruits mortality rates) and it appeared that the population growth rate and steepness distributions were robust to the choice of the CV values. α, α', β and β' are directly derived from McGurk’s estimations. Residual variances around the log-regressions are calculated with McGurk’s data to provide estimations of σ and σ' [24]. The daily mortality rate being dependent on the weight (Eqs 10 and 11), we had to estimate the daily weight of larvae and juveniles. Early life stage is split into 5 periods corresponding to specific physiological periods or to periods for which a growth curve is available. Daily weight is set as follow:
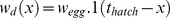
(i)

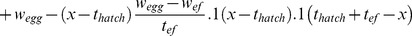
(ii)

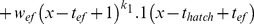
(iii)

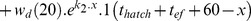
(iv)

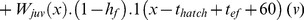
(12)where 1(*x*) is the indicator function, *i.e.* 1(*x*) = 1 if *x*>0 and 1(*x*) = 0 if *x*<0. *w_egg_* is the dry weight of the egg, *w_ef_* dry weight of the larvae at first exogenous feeding, *t_hatch_* the number of day before hatching, *t_ef_* the number of day before exogenous feeding starts, *k_1_* a power growth factor, *k_2_* an exponential growth factor, *W_juv_(x)* are the average weight of the juvenile (in grams) at date *x* after fertilization and *h_f_* the hydration factor for juveniles.

- Incubation period. Dry weight is assumed to be constant over this period at 42.8 10^−6^ g according to [52] (Table 1). Time to hatch is commonly admitted to be related to temperature [54], [55]. Incubation duration of bluefin tuna’s eggs has been measured for different temperatures and appears to be between 1.8 days at 20°C and 0.8 days at 32°C [56]; which is consistent with the relationship given by [57]. Other observations have shown that hatching occurs between 1.0 and 1.6 days after fertilization for Atlantic bluefin tuna [58] while incubation period is around 48 hours at 23°C for Pacific bluefin tuna (*Thunnus orientalis*) [59]. As the seawater temperatures encountered in the Mediterranean Sea during bluefin tuna reproduction is usually below 27°C [41], [42], minimum incubation time is set to 1 day and maximal incubation time to 2 days. Stochastic variations in duration of incubation are taken into account by assuming that *t_hatch_* is a random variable with a uniform distribution between theses 2 bounds.
- From hatching to mouth opening. During this period, only endogenous feeding is possible for the larva as the mouth is not open and the digestive system is not functional (*i.e.* yolk sac stage). Growth starts with the beginning of exogenous feeding, which coincides with mouth opening and the formation of the 1st increment on the otoliths [60]. As larvae are not able to feed, a weight loss is observed [52]. In the lack of specific information *w_d_(x)* is calculated by a linear interpolation between *w_egg_* and *w_ef_* on the number of days between *t_hatch_* and *t_ef_*. It is assumed that *w_ef_* and *t_ef_* are random variables, *w_ef_* is normally distributed with parameters based on values given in [52] to account for variability between individual weights at first feeding. The choice of bounds for *t_ef_* distribution is based on observations on various tuna species. For Atlantic bluefin tuna, first increment on an otolith is formed between 4.7 and 5.6 days after fertilization (incubation period 1.0 to 1.6), which would imply a time between hatching and first feeding of 3.1 to 4.6 days [58]. This is in agreement with *(1)* two other studies on bluefin tuna larvae, *i.e.* [61] who observed the formation of the digestive system at 3-days, and [62] who observed the onset of feeding in larvae 3 days after hatching and *(2)* several studies on related tuna species, such as the Pacific bluefin tuna [63] and the yellowfin tuna [52], [64], [65]. Considering all this information, *t_ef_* is assumed to be uniformly distributed between 2 and 4 days.
- From the first exogenous feeding to 20 days post exogenous feeding (dpef). A power growth curve is used to predict until 20 dpef with growth factor taken from [66].
- From 20 to 60 dpef. According to [61] the growth is assumed to be exponential, with *k_2_* calculated as *w_d_(t_ef_+60)* - time for which information on growth is available (see after) - reaches a dry weight determined from the growth curve used for juveniles.
- From 60 dpef to 150 dpef. We used the growth function derived from otolith readings on bluefin tuna between 600 and 1000 grams ([67]; Table 2) and took into account variability between individuals by assuming that *W_juv_(x)* was normally distributed with mean *w_juv_(x)* and 0.1 as coefficient of variation. To be consistent with the von Bertalanffy growth curve from [68] which gave the weight at the end of the year 0, dry weights between 150 and 180 days were calculated using a linear interpolation.

As mentioned above, several observations of daily mortality/survival rates have been completed and are given in Table 1 for comparison purposes.

**Table 2:** Parameters and references used for computation of steepness and population growth rate of bluefin tuna population.

Parameter		Distribution or value	Source
**Mortality rate post-recruitment**			
*A*	Terminal age	30	Restrepo et al (2009)
*µ_M(i)_*	Mean mortality rate at age i (year)	[0.49,0.24,0.24,0.24,0.24, 0.20,0.175,0.125,0.1…,0.1]	Hampton (1991)
**Mortality at age 0**			
*fh*	Mortality from laying to hatching	normal(0.5,0.05)	Rakitin et al (1999), Margulies et al 2001, Lioka et al (2000)
*a*	Daily mortality rate at unit weight (day^−1^) *w_d_* <0.00504 g	2.2 10^−4^	McGurk (1986)
*b*	Daily mortality rate scaling factor *w_d_* <0.00504 g	−0.85	McGurk (1986)
*σ*	Daily mortality rate sd *w_d_* <0.00504g	0.80	McGurk (1986)
*a'*	Daily mortality rate at unit weight (day^−1^)	5.26 10^−3^	McGurk (1986)
*b'*	Daily mortality rate scaling factor	−0.25	McGurk (1986)
*σ'*	Daily mortality rate sd	0.86	McGurk (1986)
*w_egg_*	Egg weight at fertilization (10^−6^g)	42.8	Margulies et al (2007)
*w_ef_*	Larvae weight at first exogenous feeding (10^−6^g)	normal (21.7, 4)	Margulies et al (2007)
*t_hatch_*	Incubation period (day)	uniform(0.77, 2)	Miyashita et al. (2002), Sawada et al (2005), Jusup et al (2011)
*t_ef_*	Time from hatching to first exogenous feeding (day)	uniform(2, 4)	Jenkins et Davis (1990), Kaji et al. (1996,1999), Itoh et al. (2000), Miyashita et al. (2001), Kawakami et al. (2008), Margulies et al (2007)
*k_1_*	Power growth factor 0 to 20 dpef	1.851	Garcia et al (2006)
*fl(x)*	Juvenile fork length×dpef	41.20+2.37.*x*	La Mesa et al (2005)
*w_juv_(x)*	Mean juvenile weight×dpef	1.92×10^−6^.*fl(x)* ^3.39^	La Mesa et al (2005)
*hf*	Juvenile hydration factor	0.85	Kamler (1992)
**Fecundity**			
*sr*	Sex-ratio	0.5	Tiews (1962)
*g(x)*	Proportion of mature female at age x	[0,0,0,0.5,1….1]	Corriero et al.(2003)
*Spe*	Spawning periodicity	[1], [2], [3], [4]	Lioka et al (2000), Block et Stevens (2001), Galuardi et al. (2010)
*bf*	Batch fecundity (oocytes)	normal(61.44, 48.33)	Medina et al (2002, 2007)
*N_batch_*	Number of batch	uniform(2,10)	Medina et al (2007), Jusup et al (2011)
*L_∞_*	Asymptotic size (cm)	314.90	Restrepo et al (2009)
*k*	Von Bertalanffy growth rate (year^−1^)	0.089	Restrepo et al (2009)
*t_0_*	Theoretical age at size 0 (year)	−1.13	Restrepo et al (2009)
B	Length-weight factor	1.96×10^−5^	Anonymous, 1999
C	Length-weight exponent	3.0092	Anonymous, 1999

Probability distribution functions are given for parameters defined as random variables.

### Mortality Rate from Recruitment to Terminal Age

From age 1 (calendar year) to terminal age, we considered the age-specific natural mortality vector *[µ_M(1)_….µ_M(A)_]* given by the scientific committee of ICCAT which is based on tagging experiments conducted on southern bluefin tuna, *Thunnus maccoyii* (SBT) [69]. Noise was added in mortality at age vector by randomly drawing *M(i)* in a Gaussian distribution with mean *µ_M(i)_* and CV 10%. Survival at age i *S(i)* is obtained by transforming natural mortality rates at age i with *S(i) = e^−M(i)^*.

### Absolute Fecundities at Age

#### Modeling approach

The absolute fecundity *F(i)* represents the mean contribution to spawning in number of eggs by a female at age i. Considering that reproduction might not occur every year [41], [70], we introduced the random variable *Spe* which is the interval between reproductive events (see below). *F(i)* are calculated using the following relationship:
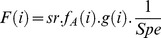
(13)where *sr* is the sex-ratio, *f_A_* the annual fecundity *i.e.* the number of eggs produced by a fish of age *i*, and *g*(*i*) the proportion of mature individual at age i. *f_A_* is given by
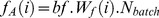
(14)where *bf* is the relative batch fecundity i.e. the number of oocytes expelled per grams of body, *W_f_* the weight of a female at age i and *N_batch_* the total number of batches during the spawning season. Note that we considered that oocytes are all hydrated and become eggs. *bf* and *N_batch_* are random variables. Weights at age *W(i)* are calculated from the fork lengths at age i *L(i)* for the East Atlantic and Mediterranean bluefin used by ICCAT, i.e. *W(i) = 1.96 10^−5^.L(i)^3.0092^* [40]. Presence of sexual dimorphism with males growing faster than females has been suggested for the Southern bluefin tuna [71] but not for Pacific bluefin tuna [72]. Regarding, ABFT, if sexual dimorphism has been reported in trap catches (larger of individuals >250 cm being mostly male, see e.g. [73]), this issue is assumed to be less acute than for other species (such as swordfish) and is not considered for ABFT stock assessment. For simplicity, we therefore assumed a single growth function. Samples of *L(i)* are drawn into normal distributions whose the mean is calculated from Von Bertalanffy growth function and CV set to 10%. which is equivalent to the variance deduced from the growth curve of West Atlantic bluefin tuna by [68]. 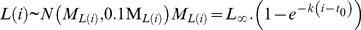



#### Data used for parameterization

The parameters of the Von Bertalanffy growth function were taken from [74].

Sex-ratio is generally admitted to be balanced for Atlantic bluefin tuna populations [41]. According to [73] and [75], median sexual maturity is reached at about 110 cm fork length which corresponds to 4 years-old females. Archival tagging information has revealed that ABFT may skip spawning in some years [70], [76]. Irregular reproduction events have been also observed for bluefin tuna in captivity conditions [53]. With no information regarding the number of years skipped for reproduction and the frequency of such event, we assume that *Spe* is independent of age and that the reproduction has the same probability (0.25) to occur every 1 (as assumed in the ICCAT assessment), 2, 3 or 4 years [77] estimated the batch fecundity per gram of body weight for Atlantic bluefin tuna, at 66.8 and 58.8 oocytes.g^−1^, respectively. Values between 126 and 56 oocytes.g^−1^ were also found in studies on fecundity of bluefin tuna and related species [51], [78]–[83]. Mean and standard deviation of *bf* distribution were calculated from the batch fecundity values given by [77], [84] which are the most recent studies using stereological analysis of ovaries. The number of batch per reproductive event has been inferred from the time spent on a spawning ground and spawning frequency. Direct observations from archival tagging indicate that a spawner might stay from 2 weeks [85] up to 39 days [86] on spawning grounds. Assuming a spawning frequency of about 1.2 days [77] and a time spent at a spawning ground of 2 weeks the total number of batches per spawning season would be at around 12. Observations of spawning events during rearing experiments indicate that this estimate corresponds to a maximum number of batch (Fauvel pers. comm.). In addition, [57] have estimated, through a bioenergetic model that the number of batch would be around 9. For all these reasons, it was assumed that N*_batch_* has a uniform distribution with lower and upper bounds respectively 1 and 10.

## Results

### Vital Rates

#### YOY mortality rate

**Figure 1:**
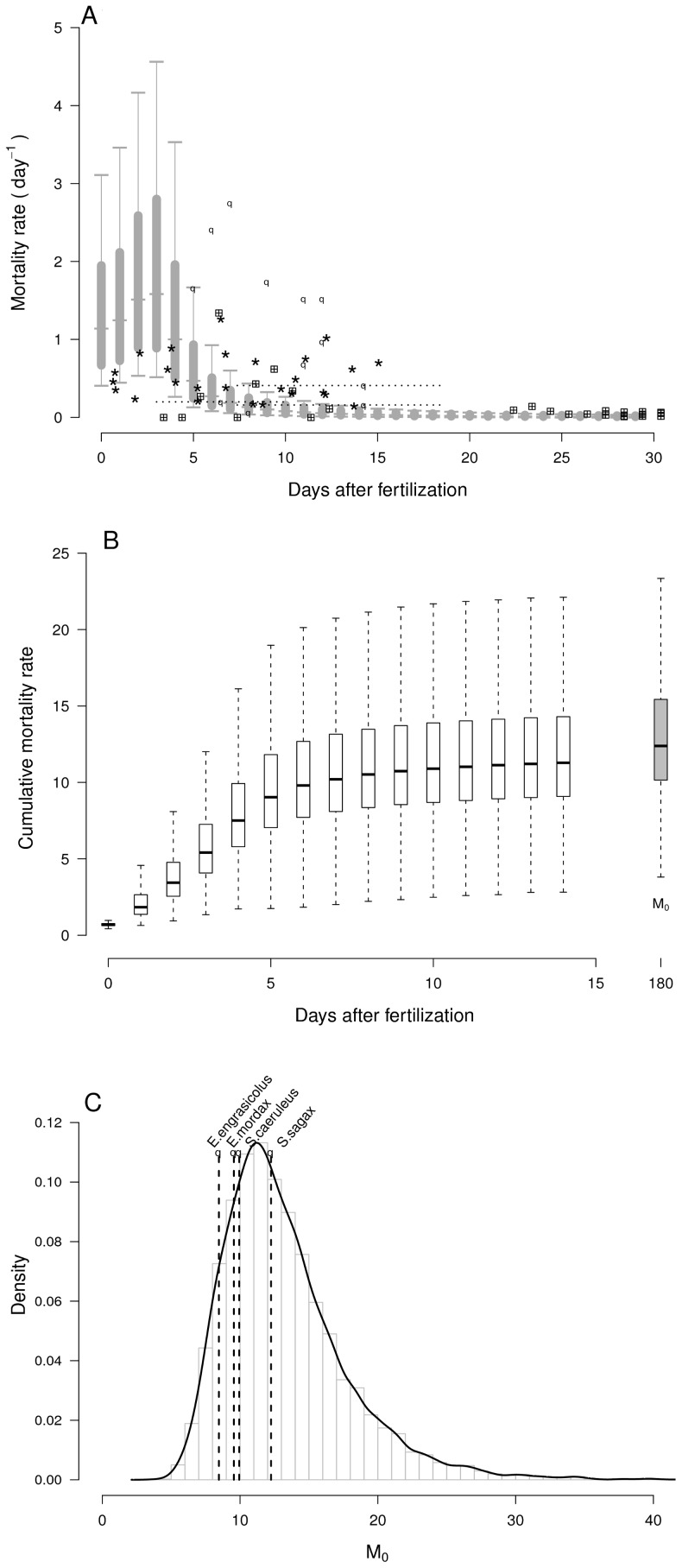
Overview of daily mortality rates and *M_0_* with comparisons to other species. (a) Distributions of daily mortality rates over 30 days after fertilization. Fine vertical grey lines represent the range between 5 and 95% quantiles, thick grey lines the interquartile range. Points represent observed mortality rates of scombrids larvae : · tunas (field observations),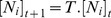 mackerel (field observations), 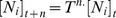 tunas (rearing observations). (b) Boxplots of cumulative mortality rates over 15 days after fertilization, comparison with cumulative mortality 180 days after fertilization. (c) Estimated distribution for bluefin tuna. Comparison on x-axis to *M_0_* of 4 small pelagic species.

During the first 4 days after fertilization, estimated daily mortality rates were above 1 d^−1^ (*i.e.* survival around 30% per day) and highly variable. Over this period of endogenous feeding, larval weight remained low which induces high mortality. Thereafter, larval growth became exponential; mean values increased from 6.5 10^−5^ to 7.2 10^−4^ grams between 5 and 10 days after fertilization. Consequently, daily mortality rates strongly decreased from 0.1 d^−1^ after 10 days to below 0.02 d^−1^ at 30 days after fertilization, and became less variable. Observed mortality rates (in rearing as well as in field conditions) were in agreement with the overall magnitude of estimated mortality rates (Fig. 1a). Cumulative mortality rates increased very rapidly as 80% of *M_0_* (180 days) is achieved after 8 days and the distribution of cumulative mortality after 15 days was almost similar to distribution (6 months after fertilization; Fig. 1b), indicating that estimates of *M_0_* (and thus its range of possible values) mainly depended on the weight estimates at earlier life stages. Unsurprisingly, the estimates of *M_0_* exhibited a skewed distribution with a median value and standard-deviation of 12.4 year^−1^ and 4.6 year^−1^, respectively (Fig. 1c). The uncertainty about the estimates of *M_0_* were substantial (*i.e.*, with 5% and 95% quantiles corresponding to values of 7.66 and 21.9, respectively). This variability led logically to a large interquartile range in the estimates of the survival of the early stages, *S_0_* that extended over several orders of magnitude. 50% of estimated values of *S_0_* range between 0.00002% and 0.004% after 180 days. Influence of specific life history traits has been investigated, especially to examine potential variability due to dependence among some random variables. Choosing *t_ef_* = 2 and *t_ef_* = 4 makes that the *M_0_* distribution have different means (of 11.4 and 15.2, respectively), but equivalent standard deviations. This indicated that the value of *t_ef_* had little influence on the variability of *M_0_* distribution but can influence its mean.

#### Fecundity

**Figure 2:**
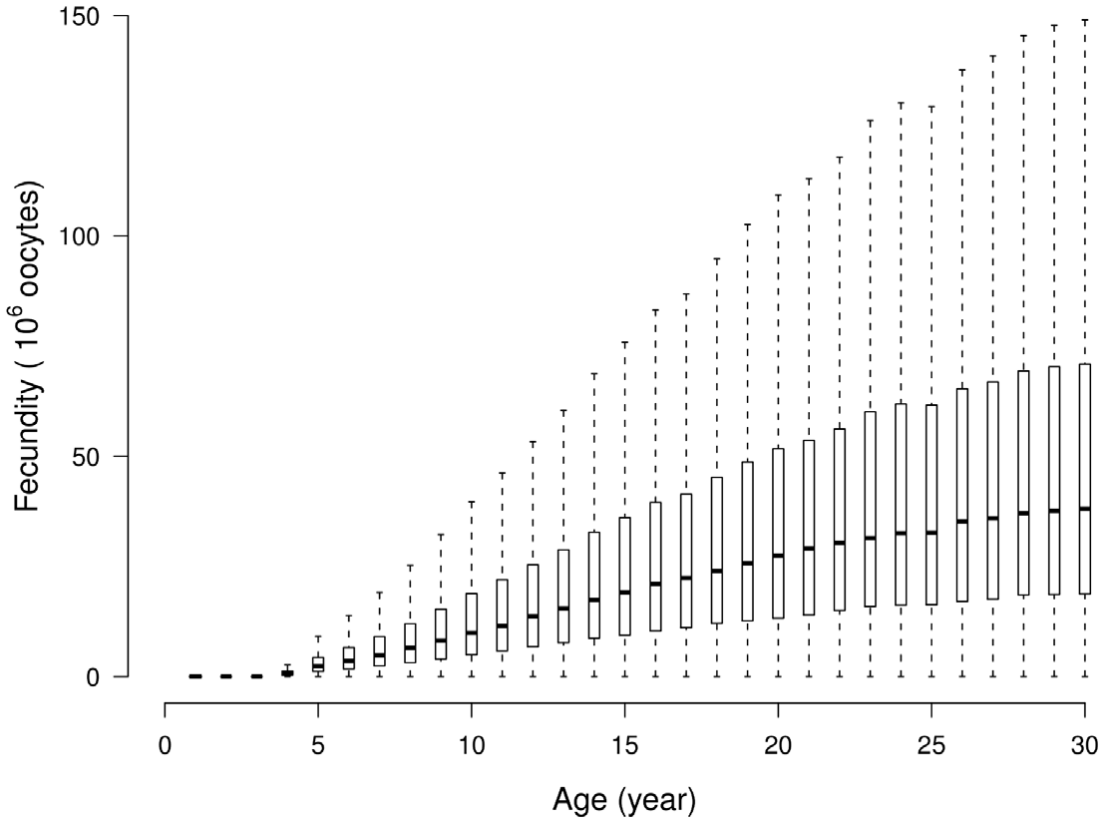
Boxplots of mean contribution to reproduction in number of oocytes.

The number of expelled oocytes per batch was estimated at about 8 million for a 10 years-old fish and at around 20 million oocytes for a 20 years-old fish. Taking into account the number of batches per year, the reproduction frequency, the mean number of expelled oocytes by a 20 years-old female would be about 32 10^6^ per year (Fig. 2). As expected, the mean value and the variance of the annual fecundity strongly increased with age. For the oldest individuals (>20 years), the median values of annual fecundity stabilized around 30 million oocytes. These distributions included a large variability with a CV close to 1 for all ages.

### Population Growth Rate

Distributions of *M_0_*, post-recruitment mortality rates and annual fecundity being estimated, it was then possible to estimate the population growth rate, *r*, using the Leslie matrix model. Population growth rate estimations extended from −0.5 to 2.5, with a median value of 0.56 (Table 3). Negative values of *r* corresponded to low fecundities or high mortality for early stages. This distribution of *r* can be thus considered as weakly informative (CV≃1), akin to a uniform distribution ranging between 0 and 1.

**Table 3:** Summary statistics of estimated demographic quantities for bluefin tuna population.

Parameter		5%	25%	50%	75%	95%	mean	sd
*M_0_*	Mortality rate at age 0	7.7	10.2	12.5	15.6	21.9	13.4	4.7
*W_bpr_*	Expected spawning biomass per recruit	401	440	470	500	549	472	44.9
*α*	Recruit per spawning biomass unit	0.00	0.03	0.55	6.00	80.4	17/08/12	74
*r*	Population growth rate	−0.20	0.15	0.54	0.95	1.49	0.57	0.53
*h*	Steepness	0.00	0.60	0.99	0.99	0.99	0.76	0.36

**Figure 3:**
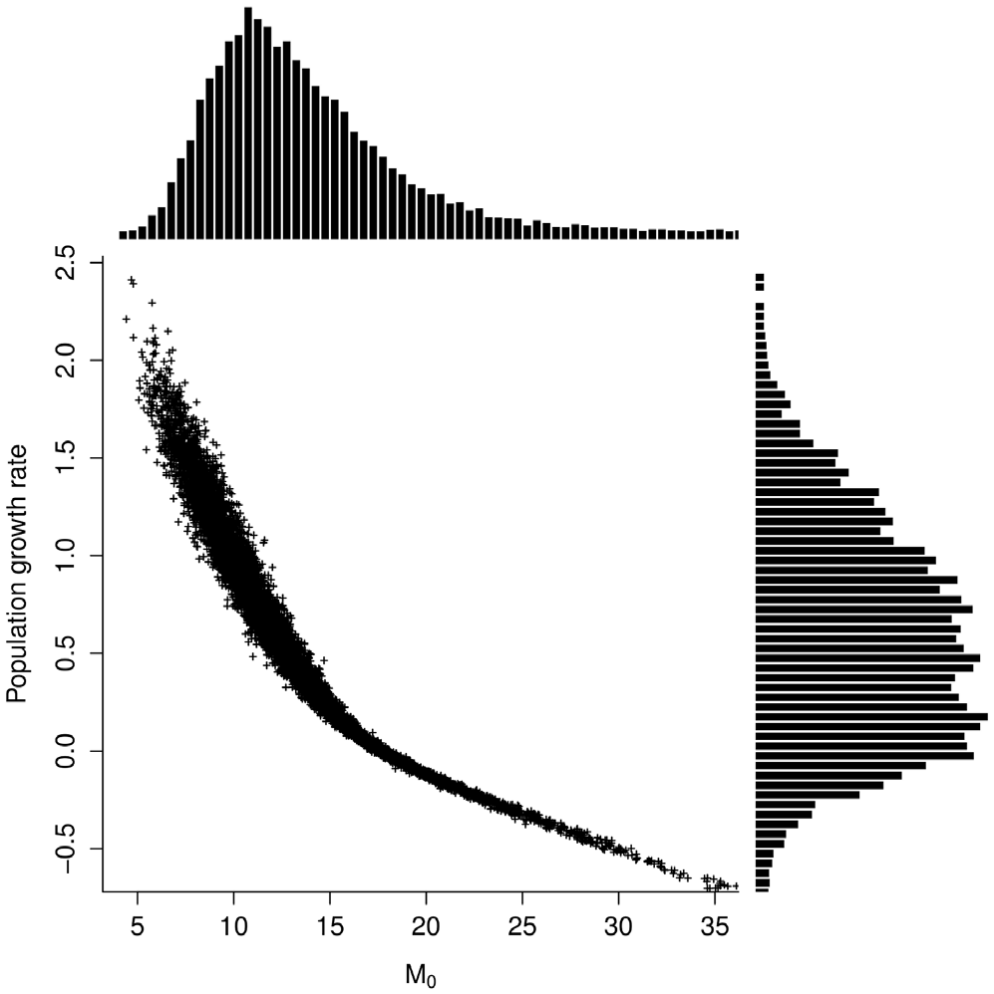
Population growth rate *r* plotted in relation to age 0 mortality rate with marginal distributions of each parameter represented along respective axes.

Since *M_0_* appeared to be a key parameter when estimating the population growth rate, a sensitivity analysis was conducted for the distribution of *r* vs. *M_0_*. Fig. 3 shows the importance of *M_0_* in population growth rate estimation. Both parameters were indeed strongly and negatively correlated (*r_spearman_ = *−*0.99*); *M_0_* values of 10 to 15 leading to *r* values between 0 and 1.

The other vital rates did not affect so strongly the *r* estimates. None of the vectors of fecundity at ages *F(4)…F(A)* showed any significant correlations with the population growth rate. When testing a modification of the population fecundity by artificially decreasing the reproduction frequency to *Rf*∼*uniform*(0.25,0.33) and the number of batches to *N_batch_*∼*uniform*(2,5), the distribution of *r* was only slightly affected: shift to the left by a value of 0.1 (with a median of 0.4 and the same standard deviation). Sensitivity of *r* and steepness to difference in age-at-maturity between the eastern and western ABFT stocks (4 *versus* 9 years old, see ICCAT 2010) has been also tested. Given this parametrization, the median value of r decreased to 0.35 which indicated an effect of the maturity schedule on the population growth rate.

Post-recruit natural mortality rates (from age 1 to terminal age) and number of age-groups were not correlated with *r* and had minor influence on the population growth rate estimates. Similar *r* distributions and statistics were obtained for a number of age-group of A = 20 and A = 30. Assuming a decrease of *µ_M(i)_* by 10%, then by 20%, successively to check the influence of natural mortality rates in *r* calculation, we found that a 10% decrease does not change r statistics. Reducing *µ_M(i)_* by 20% generated a slight shift of the population growth rates, about 0.05 to right side of the distribution.

### Steepness & Stock-Recruit Quantities

**Figure 4:**
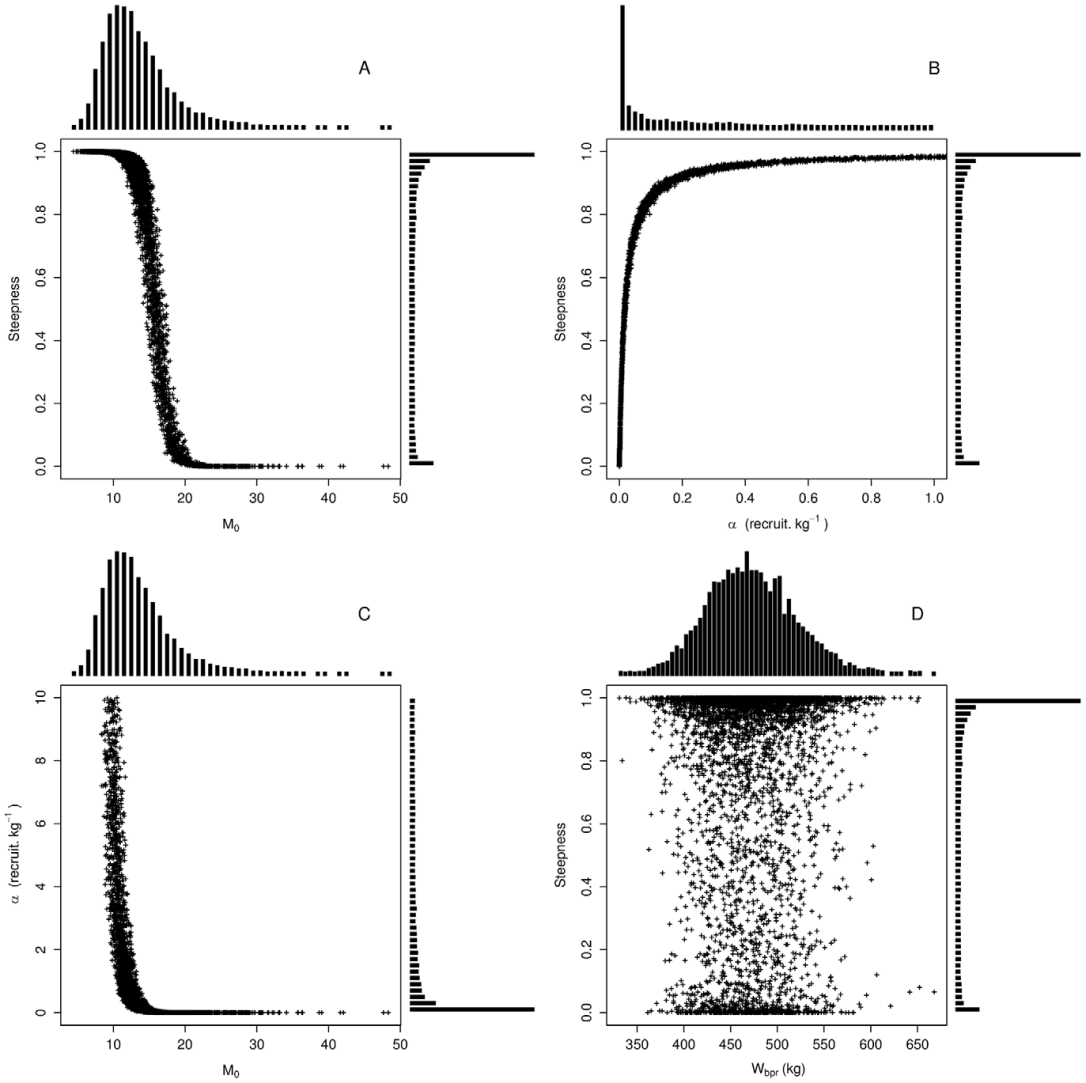
Relationships between stock-recruit quantities, steepness and *M_0_*. (a) Steepness *h* plotted in relation to age 0 mortality rate (b) Steepness *h* plotted in relation to recruits per spawning biomass α. (c) α plotted in relation to *M_0_* (d) *h* plotted in relation to spawning biomass per recruit *W_bpr_*. Marginal histogram of each parameter is represented along respective axes.

Steepness and stock-recruits quantities were estimated using the vital rates estimates described for the population growth rate. The mode of the corresponding distribution of the steepness is about 0.99 (Fig. 4b). One surviving recruit was expected to produce around 470 kg of mature biomass in unfished conditions (*i.e.* median value of *W_bpr_*, Table 3). As, *W_bpr_* depended mostly on survival and growth parameters (Eq. 5), the potential contribution to spawning stock biomass was mainly limited by mortality, which was thus restricted in our study to natural mortality. Quantitatively speaking, a 10% decrease in post-recruit mortality rates leads to a median value of *W_bpr_* of 600 kg. Note that a value of 470 kg would correspond to a fish of 284 cm long and about 23 years old, so significantly lower than the maximum length (>300 cm), maximum weight (>700 kg) and maximum age (about 40 years) of ABFT [41].

The number of recruits produced per spawning biomass kilogram (α) depicted a strong asymmetric distribution, with a few high values on the right hand side (Fig. 4b). 50% of α values range between 0.03 and 6 (Table 3). This large interval indicates that a 100 kg spawner can yield between 3 and 600 recruits per year. *M_0_* appeared again the key parameter for the calculation of the stock recruitment parameter α (Fig. 4c). Low age-0 mortality rates (*M_0_<10*) generated important number of recruits per SSB (α>1) while *M_0_>13* induced few recruits per SSB unit.

We first investigated *h* against the corresponding distributions of α and *W_bpr_* (see Eq. 4). There was no apparent relationship between the steepness and *W_bpr_* (Fig. 4d). However, *h* appeared directly related to α (Fig. 4b), as a number of recruits per kilogram of SSB >0.1 generated systematically steepness values greater than 0.8. Conversely, low steepness values were associated to a small number of recruits per unit of SSB. We further examined the sensitivity of the steepness to *M_0_* and others parameters involved in (Eq 6). As showed for population growth rate, there was a strong negative relationship between *M_0_* and the steepness (Fig. 4a). The steepness was systematically close to 0.99 for values of *M_0_*<12, while it decreased rapidly for *M_0_>14*. In addition, no significant relationship has been found between *h* and *F_sim_* or *B_sim_*. The potential influence of the fecundity on the steepness had been checked by decreasing the reproduction frequency and the batch number as described for *r*. In such case lower estimates for α were obtained (mean = 11.15 and median = 0.46) but *h* distribution remained unchanged.

### Role of M_0_

**Figure 5:**
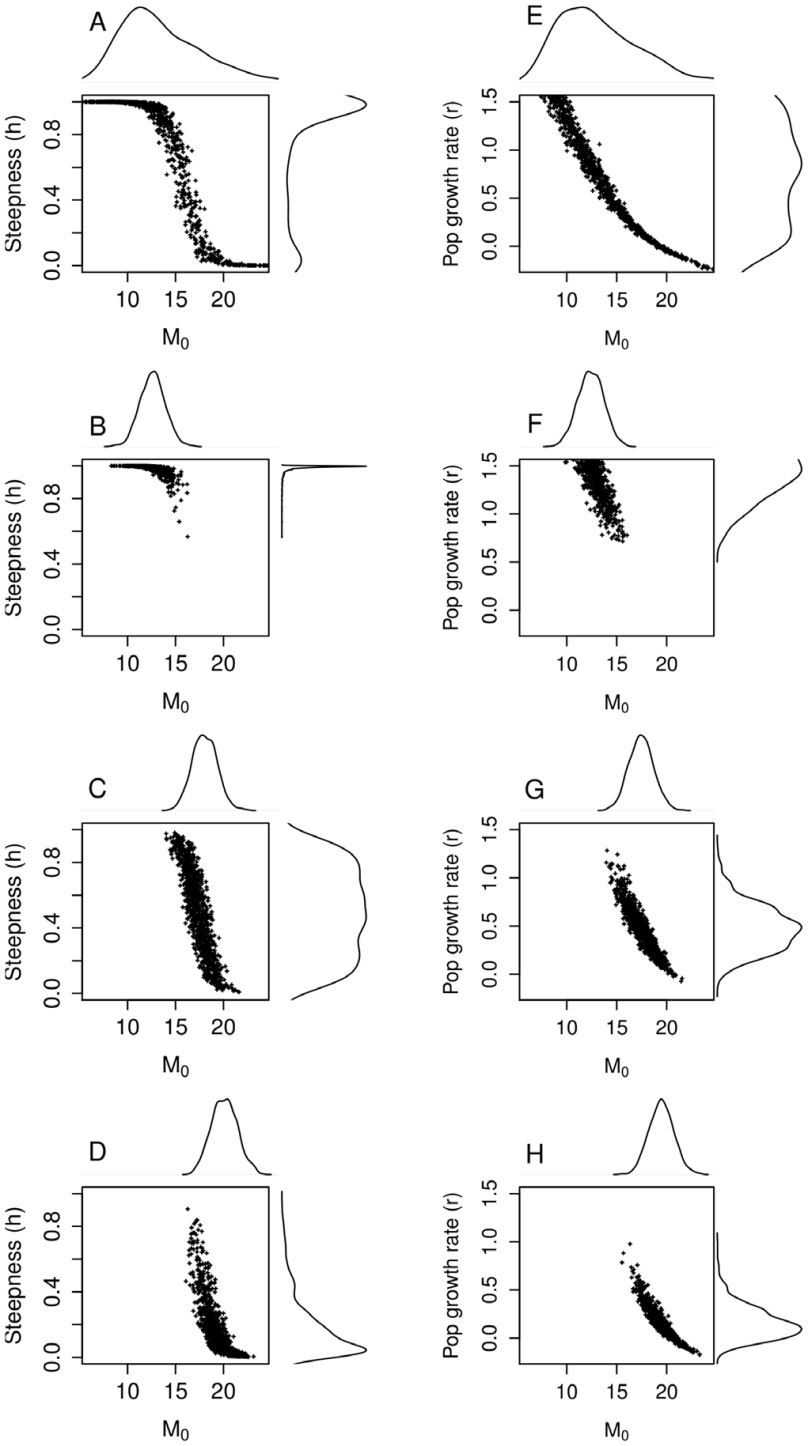
Steepness *h* (left panel) and population growth rate *r* (right panel) plotted in relation to different distributions of *M_0_* (age 0 mortality rate). (a-e) the whole distribution of *M_0_* (mean 13.4 and CV 35%) (b-f) a simulated Gaussian distribution with mean equal 12.5 (median value of the whole distribution) and CV = 10% (c-g) Gaussian distribution with mean equal to 18.5 and CV = 10% (d-h) Gaussian distributions with mean equal to 21.5 and CV = 10%.

For illustration purposes, *r* and *h* were re-calculated using informative distributions of *M_0_*. Three different Gaussian pdf were simulated with mean 12.5, 17.5 and 19.5 year^−1^ respectively and a CV of 10%. Precise distributions of *M*_0_ induced more informative distributions for *r* and *h*, but with different means and modes (Fig. 5). The modes of the steepness distributions ranged from 0.9 in the case b to 0.2 in the case d while the modes for the population growth rate varied from 1.1 to 0. Additionally, the steepness displayed a non-informative distribution for M_0_ in the interval [15], [20] (Fig. 5c). This simple simulation exercise clearly showed how much sensitive are *r* and *h* distributions to *M_0_*.Note that the high mortality scenario (*i.e.* Fig. 5 d-h) led to unrealistic distributions of *r* and *h.*

The above results were further robust to potential dependence among the different life history traits variables. Correlations of 0.7 and 0.9 between *M_0_* and total fecundity did not reduce the variance of *r* distribution and did not change this of *h* (for a correlation of 0.9 between *M_0_* and total fecundity, *r_spearman_ is* −*0.99* between *M_0_* and *r* and between *M_0_* and *h*). This result confirms that *r* and *h* distributions are primarily driven by the *M_0_* distribution.

### Corrected Bayesian Priors

**Figure 6:**
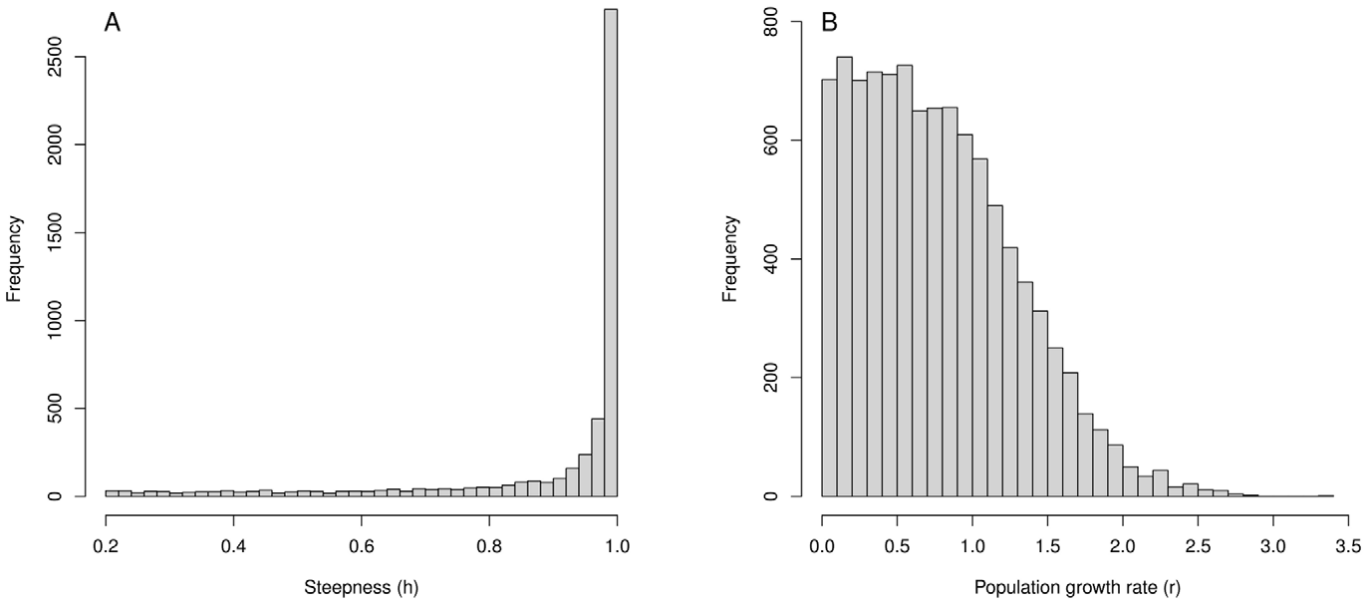
Distributions of the steepness (*h*) and the population growth rate (*r*) obtained with an acceptance-rejection procedure to limit *r* range to [0,+∞]and *h* range to [0.2, 1].

Values of *r* and *h* lower than 0 and 0.2, respectively, referred to biological situations where the bluefin population would be in decline and are thus not consistent with the basic assumptions of the biomass dynamic model and the Beverton & Holt SR model. As illustrated by Fig.3 and 4a, high values of *M_0_* (>18) were responsible for both negative values of *r* and values of *h* <0.2 (some values that make sense within the estimation using the Leslie matrix model). A procedure was then implemented to reject all random drawings for which *r<0* or *h<0.2*, to obtain prior distributions for *r* and *h* usable in a biomass dynamic or Beverton & Holt models. In that case, *h* distribution still displayed a great proportion of values close to 0.99 while the *r* distribution had a greater mean and median than in the initial case, but still an important CV (Fig. 6a,b and Table 4).

**Table 4:** Summary statistics of estimated demographic quantities for bluefin tuna population obtained with the acceptance-rejection procedure.

Parameter		5%	25%	50%	75%	95%	mean	sd
*M_0_*	Mortality rate at age 0	7.4	9.8	11.6	13.8	16.4	11.78	2.74
*W_bpr_*	Expected spawning biomass per recruit	400	440	470	500	548	471	45
*α*	Recruit per spawning biomass unit	0.01	0.14	1.25	8.45	99	22	86
*r*	Population growth rate	0.07	0.34	0.70	1.12	1.74	0.77	0.53
*h*	Steepness	0.38	0.88	0.99	0.99	0.99	0.89	0.20

## Discussion

The use of life history traits appears appealing for the elicitation of priors for demographic parameters and some relationships may be elaborated between vital rates, steepness and population growth rate. However, the prior elicitation of such parameters requires estimates of key life history traits over the whole life cycle of the species. Large uncertainties are inherent to such demographic approaches on most marine teleost species principally because of intrinsic variability and partial knowledge of mortality schedules and reproductive ecology. We have addressed this point by considering almost all life history traits as random variables which were assigned probability distribution reflecting these uncertainties. Furthermore, sensitivity of population growth rate and steepness to most of life history traits has been checked. Apart from sensitivity of population growth rate to age at maturity (see [87]), influence of reproductive ecology and post-recruit natural mortalities on steepness and population growth rate is limited.

Complex biological processes have been modeled by functional relationships that usually used in fisheries science or marine ecology (such as the Von Bertallanffy growth curve). These simplifying assumptions may have influence our results, but we could hardly take into account for alternative models or dynamics for key biological processes (such as mortality, growth, maturity) because this would have induced a huge number of additional simulations. Therefore, we only kept simple and widely-used functions and relationships.

After a detailed study of the entire life cycle of the bluefin tuna, we conclude that *M_0_* is the critical biological parameter to precisely estimate the steepness and the population growth rate. In other words, a reasonably informative distribution of *r* would imply that *M_0_* is known precisely. As *M_0_* is highly variable, this results in poorly informative priors of *r*. Our findings showed that the distribution of *r* is more variable than suggested by previous studies, which did not deeply investigated basic biological parameters, such as natural mortality of early life stages. [6] used a prior distribution of r ranging from 0.13 to 0.48 (10% and 90% quantiles, respectively) while *a priori* estimates provided by [28] for yellowfin tuna and albacore populations were at 0.2 and 0.4, respectively. For the steepness, [24] noted a mode around 0.9 for the Southern bluefin tuna population while through a meta-analysis [21] estimated this parameter at 0.52 for Scombridae and at 0.88 for swordfish (*Xiphias gladius*), by basing their estimates on stock assessment estimates. In our study, the mode of the steepness distribution is very close to 0.99 because of a large proportion of *M_0_* values <15. The low proportion of steepness values <0.2 (12% of the initial sample of *M_0_*) were generated for *M_0_>18*. Although, Pacific bluefin and ABFT have different maturity schedules, the difference between our estimate of h and this by [24] for a similar species (*i.e.* Pacific Bluefin tuna) is likely to come from differences in M_0_ distribution. Our results have clearly showed that the distribution of *M_0_* does really matter in h estimates. Growth functions for eggs and larvae are different in [24] and this study, which may have caused differences in early mortality rates. The estimate of *M_0_* is based on an empirical "mortality-size" relationship described by many authors [47], [88], [89]. Indeed, there is no general process-oriented modeling approach for estimating mortality rates of young stages of bony fish, although some new approaches based on heavy sampling effort have been recently developed, see e.g. [90] for anchovy. As a process-oriented modeling approach for the natural mortality of bluefin larvae has not yet been proposed, this empirical relationship for *M_0_* appears then as a practical approach. McGurk's relationship is based on a dataset gathering various bony fish species which however display rather similar early-life strategies. As mentioned all the parameters (e.g. incubation and yolk sac period duration, larval growth rate) involved in the *M_0_* calculation are random variables, so that we could have kept all sources of uncertainties attached to the biological and ecological processes of interest. We used additional information from other species when we had no relevant information for ABFT and only from close tuna species (such as southern or Pacific Bluefin tuna and yellowfin tuna). This approach remains, to our knowledge, pertinent as tunas species exhibit similar reproductive biology and behavior. The values of the different life history traits variables that we used came from studies that did not take into account for potential correlations among these variables and this could result in over-estimating the final variances. The means of *M_0_*, *r* and *h* distributions were affected by different values of some early-life history traits, especially *w_ef_* and *t_ef_*. As they were defined as random variables, the variability of these quantities is incorporated in the final distributions. However, we showed that the variability in *M_0_*, *r* and *h* distributions is poorly affected by the use of different values for *t_ef_* and thus by the potential dependence between life history traits variables.

**Figure 7:**
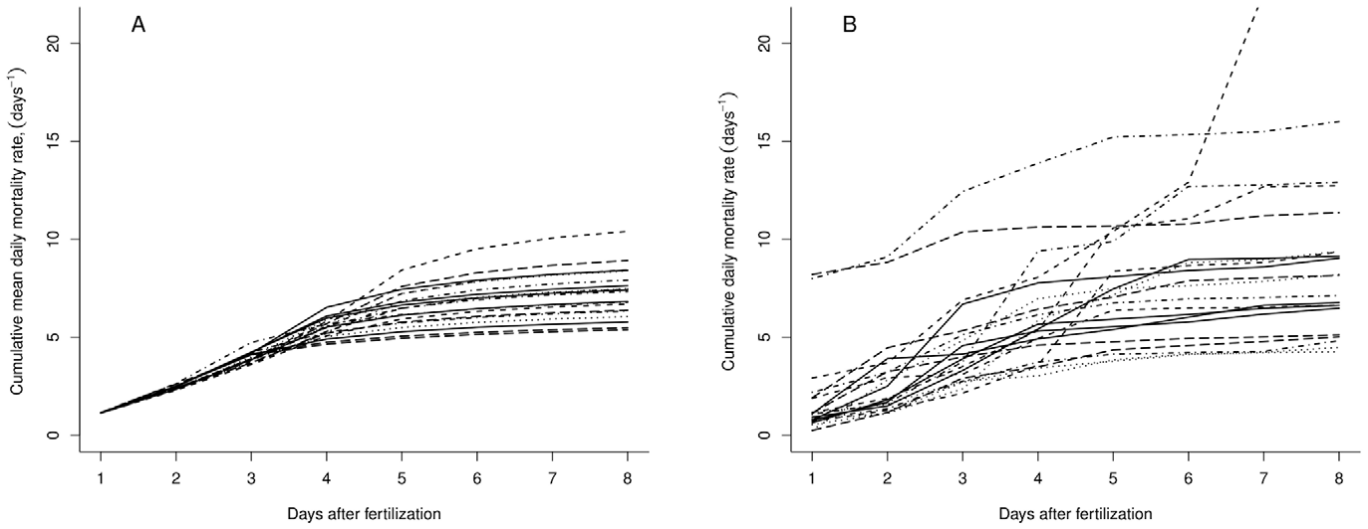
Comparison between cumulative early mortality including or not variance of McGurk's log-regressions. (a) Sample cumulative mean mortality rates *µ_Md(x)_* from 1 to 8 days after fertilization., *µ_Md(x)_ = a+b.log(w_d_(x))* for wd<0.00504 and *µ_Md(x)_ = a'+b'.log(w_d_(x))* for wd>0.00504 (b) Sample cumulative mortality rates *M_d_(x)* from 1 to 8 days after fertilization, taking into account Z_σ_ and Z_σ'_ the estimated variances around McGurk's log-regressions.

Our results are consistent with available estimates for small pelagics, see Fig. 1c for a comparison with [90]–[93]. Furthermore, our estimated intrinsic variability in larval mortality is in agreement with the rare *in situ* estimates carried out by [46] who found, at sea, very high day-to-day variations in *M_0_* of Pacific bluefin tuna (ranging from 0.06 to 2.75 d^−1^). The large variability in *M_0_* distribution is mostly due to the standard deviation re-calculated from the McGurk’s allometric relationship. There is indeed a radical difference in the dispersion of the cumulative mortality rates from 1 to 8 days - and consequently on *M_0_* - if the residual variance in McGurk's log-regression is taken into account or not (Fig. 7). By considering daily mortality rates as random variables, we generated a larger variability in *M_0_* that is then transmitted in the demographic quantities of interest.

This large variability in *M_0_* is in agreement with our biological knowledge and the extensive past and present literature on the dynamics of fish recruitment. Since Hjort [35], [36] year class strengths are known to be primarily determined by the survival of early life stages, especially during the very first weeks of life (*i.e.* the so-called critical period). Although it has been established that the strength of a year class can be set later in the ontogeny - e.g. juvenile stages see [94], [95]- various examples demonstrate that starvation is the main factor responsible for larval mortality [96], [97] and that the exposition to starvation is highly variable in space and time [96]. Consequently, a large part of uncertainty in *M_0_* can be considered as natural variation (*sensu* [98]), which makes the true value of this parameter hardly predictable. The stochastic aspect of the process partially results from the necessary match between the simultaneous occurrence of fish larvae and planktonic food availability, also known as the match-mismatch hypothesis [99] which has been successively described in various marine ecosystems (see e.g. [100]–[102]). Stochasticity may also result from the fact that fish larvae need dense concentration of proper-sized food which are attained when ocean is calm and when there is a stable mixed layer, otherwise larvae can be drifted away proper areas and die from starvation [97], [103]. Those hypotheses have further developed through the optimal environmental window [104], [105], and ocean triad [106] who suggested that the recruitment success in upwelling areas is mainly determined by three variables : the planktonic production, the turbulence and the retention. Variations in natural mortality is not only due to abiotic (environmental) events, but also to biotic processes. Predation by other fish species or jelly fish, cannibalism and competition resulting from food and/or habitat limitation can also strongly affect the mortality (and its variability) of eggs and bony fish larvae (see [107]–[111]). These processes could induce density-dependent mortality/growth, which can reduce the variability induced by stochastic factors during the early stages [94], [112] but also generate cycles and/or long-term fluctuations in fish stocks [113]–[116]. In the present analysis, we did not attempt to differentiate the density-dependent and density-independent mortalities because the allometric relationship that we used directly integrates both types of mortality. Other aspects of the life cycle of the bluefin tuna have still to be studied. Our knowledge about the reproductive biology is still incomplete and does not allow us to precisely estimate the mean annual fecundity of a female. For instance, the lack of information on bluefin reproductive behavior led us to make rather strong assumption about the number of batch per year and the spawning frequency. Nonetheless and surprisingly, uncertainties on the reproductive biology barely affect *r* and *h* which are primarily dependent on the natural mortality at age-0.

The major consequence of this high variability in *M_0_* is the difficulty to limit *a priori* the range of possible values for the steepness and for the intrinsic growth rate of ABFT population, and possibly for most of the pelagic bony fish stocks which display a similar early life history trait and reproductive biology. The operational Bayesian prior that we finally proposed (Fig.6) is weakly informative compared to the currently admitted priors for *r,* which makes the *c*onsequences on stock assessment methods for tunas and similar species potentially important.

For age-structured models used for tunas stock assessment (e.g. VPA-ADAPT [10]), the SR relationship is generally used to determine biological reference points and to predict the possible status of the stock under various fixed catch scenarios. ABFT in the East Atlantic and Mediterranean is a specific case as *F_0.1_* was used as proxy for MSY based reference points, and projections were based on historic recruitments. However, such relationships are most often difficult to directly fit on recruitment and SSB data because of the lack of points at low biomass. Therefore, it is often necessary to assume a value for the steepness [14]. Despite high natural variations in *M_0_*, the distribution of h is surprisingly informative and peaks at 0.99. This results that mostly comes from the highly nonlinear relationship between *M_0_* and *h* (as depicted by [24]) would mean that the spawning stock biomass is not sufficient to predict accurately the recruitment success. Note that such output is not odd from a biological viewpoint, regarding the very high fecundity of tuna (a large female can indeed produce several millions of eggs per spawning season, so that it may be difficult to detect any biomass/recruitment relationship et the population level. Although such a relationship has been assumed in this paper, further investigation are needed to disentangle environmental from parental effects on the recruitment.

Regarding the biomass dynamic model, its formulation within a state-space modeling framework is relevant, as it allows to separate process and observation errors [1]. However, it requires the use of informative priors for the population growth rate the carrying capacity (these two parameters being further highly correlated), the calculability, or the errors terms. Our study showed that a prior based on life history traits for the population growth rate is weakly informative because of high variability in *M_0_*. As stated by [117] productivity varies between tuna stocks because of differences in life history traits, such as age-at-maturity (e.g. the Western Atlantic bluefin should be less productive than the Eastern BFT as hypothesized by [118]). However, intrinsic natural variability in *M_0_* maintains important level of uncertainties in r prior distributions, so that it becomes harder to detect differences in productivity between species.

Most of fisheries stock assessment methods rely on numerical models that have been continuously increasing in complexity [119]. However, the use of these complex statistical stock assessment models has several drawbacks: (i) they can only be applied on “data-rich” stocks, (ii) they are often overparameterized and could thus easily lead to non-robust results and (iii) they don’t assess the effects of uncertainties in some key processes on the performances of the different management options. To circumvent this last point, there is an increasing effort in developing Management Strategy Evaluation (MSE, see e.g. [120]–[122]). Doing so, [123], [124] investigated the robustness of the current stock assessment procedure of bluefin tuna with respect to uncertainty about the true population dynamics (especially long-term variations in carrying capacity or migration patterns). MSE are, however, highly complex and also mostly limited to “data-rich” stocks. This growing complexity should thus not overshadow that a detailed and sophisticated modeling approach is not absolutely necessary to properly manage exploited fish stocks [125], [126]. Studies based on empirical indicators and on simple empirical management procedure have been developed over the last decade and have shown to be as or more powerful than more complex approaches (see e.g. [127]–[131]).

Eliciting informative priors for quantitative stock assessment methods from biological information model have limits in the current state of knowledge. Better estimates of reference points in a Bayesian framework need better understanding of early life history traits to elicitate more informative prior and the use of more reliable data *e.g.* improvement of index of abundance/recruitment used for steepness estimates [132]. In parallel, complementary and alternative approaches such as empirical indicators should be investigated for species like bluefin tuna.
